# Psychometric properties of the Italian version of the staff attitude to coercion scale: an exploratory factor analysis

**DOI:** 10.3389/fpsyt.2023.1172803

**Published:** 2023-05-24

**Authors:** Paola Venturini, Giulia Bassi, Silvia Salcuni, Georgios D. Kotzalidis, Carla Ludovica Telesforo, Eleonora Salustri, Manuela Trevisi, Valentina Roselli, Lorenzo Tarsitani, Vittorio Infante, Cinzia Niolu, Gianmarco Polselli, Tommaso Boldrini

**Affiliations:** ^1^Department of Psychiatry, Rome ASL Roma 1, Rome, Italy; ^2^Department of Developmental Psychology and Socialization, University of Padova, Padua, Italy; ^3^Department of NESMOS, Faculty of Medicine and Psychology, Sapienza University of Rome, Sant’Andrea University Hospital, Rome, Italy; ^4^Department of Human Neurosciences, Faculty of Medicine and Dentistry, Sapienza University of Rome, Policlinico Umberto I, Rome, Italy; ^5^U.O.C. Psichiatria e Psicologia Clinica, Policlinico Tor Vergata, Università di Roma Tor Vergata, Rome, Italy

**Keywords:** coercive measures, attitudes, test adaptation, compulsory treatment, involuntary admissions, exploratory factor analysis

## Abstract

**Aims:**

The current study aimed to validate the Italian version of the Staff Attitude to Coercion Scale (SACS), which assesses mental health care staff’s attitudes to the use of coercion in treatment.

**Methods:**

The original English version of the SACS was translated into Italian, according to the back-translation procedure. Subsequently, it was empirically validated by performing an exploratory factor analysis on a sample of 217 mental health professionals (Mean = 43.40 years, SD = 11.06) recruited form Italian general hospital (acute) psychiatric wards (GHPWs), with at least 1 year of work experience (i.e., inclusion criteria).

**Results:**

Results confirmed the three-factor solution of the original version for the Italian version of the SACS, though three items loaded on different factors, compared to the original. The three extracted factors, explained 41% of total variance, and were labeled similarly to the original scale and according to their respective item content, i.e., *Factor 1* “Coercion as offending” (items: 3, 13, 14, and 15), *Factor 2* “Coercion as care and security” (items: 1, 2, 4, 5, 7, 8, and 9), and *Factor 3* “Coercion as treatment” (items: 6, 10, 11, and 12). The internal consistency of the three-factor model of the Italian version of the SACS was assessed through Cronbach’s α and yielded acceptable indexes, ranging from 0.64 to 0.77.

**Conclusion:**

The present findings suggest that the Italian version of the SACS is a valid and reliable tool that can be used to assess healthcare professionals’ attitudes toward coercion.

## Introduction

Coercion is one of the principal ethical issues in the psychiatric context worldwide (1–3). Restraint, forced medication, and seclusion are daily routine treatments for many patients, both for safety and treatment reasons, with some differences according to the legal regulations of the various countries. However, these procedures, apparently aimed at benefiting patients, are a source of much controversy and risk for malpractice, abuse and death of such patients (4–6).

As to the proportion of psychiatric patients subjected to coercion, studies indicate wide variability among countries and among different contexts within the same country, with wide variations in the type of coercion measures adopted and their duration (7). A European study showed that about 38% of psychiatric patients receive coercive treatments (8), and similar proportions were found in the US (9). Considering the high risks and costs of coercion for both patients and staff, various attempts at reducing or avoiding coercion were made and proposed [e.g., National Association of State Mental Health Program Directors (NASMHPD), (10); Substance Abuse and Mental Health Services Administration (SAMHSA), (11, 12)]; however, their complete abolition seems currently impractical (7). Several risk factors for the application of coercive interventions have been identified, such as socio-demographic, psychopathological and clinical patient characteristics, poor education of staff, de-escalation techniques, and ward facilities (8, 13–20). However, results from the above-mentioned studies are mixed and cannot explain the observed wide variability in terms of incidence of patients undergoing coercion. For this reason, mental health professionals’ attitudes have been examined to explore their impact on coercive interventions (21–23).

The attitude toward something takes into account several components, some of which are explicit, such as cognitive and behavioral ones, and others implicit, like affective and unconscious ones, which all influence clinicians’ decisions (24).

Investigations performed in the last 30 years aimed to better analyze staff attitude toward coercion measures. The first questionnaire was developed by Klinge in 1994 and consisted of 40 items, which explore staff attitude toward seclusion and isolation. The author concluded that staff mostly preferred isolation rather than restraint (25). Almost 10 years later, Alem et al. (26) developed an elaborate questionnaire regarding the evaluation of the staff attitudes and found that attitudes toward using coercion could be placed on a continuum from ethical through neutral to an unethical view of it in mental health care. Finally, Wynn (27) developed a questionnaire investigating the frequency of coercive interventions in the past year in a psychiatric ward, the staff’s opinion of their current use, the reasons why the coercive measures are used and the effect they have. In this study, the author found that mental health professionals who approved coercion had been more often victims of previous physical aggression by the patients.

More recently, inspired by Alem’s et al. research, Husum et al. (28) developed a self-report questionnaire, namely the Staff Attitude to Coercion Scale (SACS), in order to explore and assess staff attitudes toward coercion practices in mental health care. The SACS consists of 15 statements on the use of coercion, on how participants think about it, and whether they consider coercive interventions to be necessary (28, 29). The questionnaire was initially developed in Norwegian and then translated into English, showing good reliability, validity and feasibility (28).

Using factor analysis, three underlying dimensions of staff attitude to coercion emerged.

The first scale, *“coercion as offending,”* reflects a negative attitude toward coercion. Items within this dimension endorse the view that coercion is potentially harmful, it offends patients, and can violate the relationship between the caregiver and the patient, thus it should be avoided or reduced. The second scale, *“coercion as care and security,”* represents a pragmatic attitude in which coercion is necessary for care and security (of both patients and staff). Items within this dimension justify the use of coercion for security reasons and for ensuring patient care. According to this view, the use of coercion is not considered positively, to be pursued, or desirable, but rather something necessary for safety and security reasons. The third scale, *“coercion as treatment”* reflects a positive attitude toward coercion and consequently considers it as a proper therapeutic intervention. Its items endorse a positive view of the use of coercion.

As far as we know, the SACS is currently the only validated instrument measuring staff attitudes to coercion. It has been translated into several languages, indicating a potential for cross-cultural applicability (30), and has been adopted in several geographical and cultural contexts (31–34).

Considering the lack of current data about the extent of coercive measures in Italy, the absence of Italian studies focusing on staff attitudes toward coercion, and the inconsistency of international findings, we performed an exploratory factor analysis (EFA) with the purpose to validate the Italian version of the SACS and to explore its latent dimensions in a sample of Italian staff members working in psychiatric wards. We decided to conduct an EFA (rather than, for example, a Confirmatory Factor Analysis) to detect the most suitable factorial structure within an Italian sample, also considering the heterogeneity of factorial structures found in previous SACS validation studies—e.g., one-factor (24), three-factor (28, 35), and four-factor (36) structures.

## Materials and methods

### Participants

A total of *N* = 217 Italian staff members (57.6% females) of general hospital psychiatric wards (GHPWs) aged between 26 and 73 years (*Mean age* = 43.40, *SD* = 11.06) participated in this study. The sample consists of *n* = 98 nurses (45.2%), *n* = 90 psychiatrists (41.5%), *n* = 8 social-healthcare operators (3.7%), *n* = 3 hospital technicians (1.4%), *n* = 3 psychologists (1.4%), *n* = 3 social workers (1.4%), and *n* = 12 individuals who did not specify their role. The unbalance between nurses/psychiatrists and other professionals mirrors the national distribution of staff in public mental health departments [see (37–39)]. Overall, the staff members had an average of 9.40 years (*SD* = 10.07) of work activity in psychiatric wards and none of them had less than 1-year experience.

### Procedure

#### Design

Two Italian psychiatrists (PV and GDK) with experience in the field translated and adapted the 15 items of the original English SACS version (28) into Italian regarding sentence content and wording, as reported in Supplementary Table 1. More specifically, each item was back-translated by an independent and bilingual translator (PJ), who did not have access to the source text. Subsequently, a revision of the back-translation was carried out by two Italian translators (PV and GDK) who compared the back-translation with the source text, identifying discrepancies and discussing with the back-translator whether any changes needed to be made. Several adjustments were carried-out after the first pilot administration (we first conducted an inquiry among members of our ward’s staff and invited compilers to review their scores after receiving adequate explanation) because some items were misunderstood by the staff, for example some nurses did not understand the term “insight,” which was thus replaced with the more comprehensible “awareness of illness.” The final Italian SACS version was submitted to the original developers who approved the version and granted the right to use it in general and for conducting the present study.

#### Data collection

Data were anonymously collected between September 2022 and January 2023 by sending an online survey to the e-mail addresses of staff members working in eight different GHPWs in the Lazio region (Italy). We involved all the staff members working in the GHPWs who accepted to participate and sent the survey to 300 professionals. Of these, 217 accepted to fill-in the online questionnaire (response rate 72.33%). Before starting the survey, participants were asked to read information about the purpose of the study and provide informed consent. Respondents who reported less than 1 year of work experience in a psychiatric ward (inclusion criteria) were to be later removed from the sample. Respondents were required to answer all questions in order to conclude the survey. The study received institutional approval and complied with the 1964 Declaration of Helsinki and subsequent amendments (World Medical Association 64th General Assembly, Fortaleza, Brazil, October 2013).

### Measures

The survey included (1) questions related to age, sex, work role, and years of work activity in psychiatric wards in order to describe the reference sample, and (2) items from the SACS in order to explore its factorial structure.

The Staff Attitude to Coercion Scale [SACS; (28)] has been described in the Introduction section, along with the details of its factorial structure. Briefly, it is a 15-item self-report scale assessing the cognitive dimension of mental health professionals’ attitudes toward coercion. Each item is scored on a 5-point Likert scale from 1 “totally disagree” to 5 “totally agree.” Cronbach’s α reliability coefficient of the original SACS, upon computing the factor analysis, ranged from 0.69 to 0.73.

### Data analysis

According to standard guidelines (40), the sample size (*N* = 217) is considered appropriate for conducting EFA on the 15-item SACS. More specifically, the rule-of-thumb is to reach a number of subjects that is at least equal to 10 times the number of items (41).

The statistical analyses were performed using R (42) and by means of the “*lavaan*” (43) and “*psych*” (44) packages. Prior to performing EFA, we conducted a few preliminary analyses. The main descriptive statistics (i.e., mean, standard deviation, skewness) as well as the distribution of item responses (i.e., frequencies) were evaluated. We further calculated the Kaiser-Meyer-Olkin Measure of Sampling Adequacy (KMO-MSA) to determine whether the use of factor analysis on the present dataset was feasible. KMO-MSA values equal to or greater than 0.90 indicates marvelous sampling, around to 0.80 meritorious sampling, around to 0.70 middling samplings, while equal to or below 0.50 suggests unacceptable sampling for computing an EFA (45). Furthermore, Bartlett’s sphericity test, whose null hypothesis is that the measured variables’ correlation matrix is the identity matrix, was conducted to assess whether a data reduction technique such as EFA is meaningful for the present dataset. An oblique rotation (Oblimin) was applied to take into account possible correlations between factors. We generated Cattell’s scree plot of *eigenvalues* to preliminarily estimate the number of relevant factors that describe the data.

Based on the above-mentioned preliminary analyses and on extant literature (24, 29, 35, 36), an EFA was performed to examine the SACS latent dimensions by considering up to four-factor models and by relying on maximum likelihood (ML) estimation, which is suitable for ordinal data (46). The most plausible model was identified by leveraging on the following criteria. First, the Bayesian Information Criterion (BIC) was adopted to compare the above-mentioned models, according to the rule by which lower values indicate a better model (47). Second, the variance explained by the model identified by the BIC should reach a reasonable level of at least 40%. Third, factor loadings should be at least 0.40 (48) and their content should be coherent with the meaning of each factor. Furthermore, the factor correlation matrix was computed to investigate relationships between factors.

Upon identifying the most plausible model, its internal consistency was assessed using Cronbach’s α computed on the polychoric correlation matrix (49); α values of equal to or above 0.60 were considered to be acceptable, those below 0.60 unacceptable (50, 51).

## Results

### Descriptive statistics

Table 1 shows that, overall, the 15 items of the Italian version of the SACS are weakly skewed, fluctuating around 0, thus suggesting that the item response distribution is symmetric and therefore more reliable. Indeed, these data support the use of the ML estimator in conducting EFA.

**TABLE 1:** Descriptive statistics and item response distribution of the Italian version of the SACS.

	Frequency (%)					Mean	SD	Skewness
	1	2	3	4	5			
SACS1	1.4	6.9	20.3	47.0	24.4	3.86	0.91	-0.71
SACS2	1.4	5.1	14.7	57.1	21.7	3.93	0.83	-0.97
SACS3	6.9	24.9	29.5	26.7	12.0	3.12	1.12	-0.02
SACS4	27.2	38.7	17.5	11.5	5.1	2.29	1.14	0.73
SACS5	5.1	12.9	22.1	43.8	16.1	3.53	1.07	-0.62
SACS6	41.9	31.8	18.9	6.0	1.4	1.93	0.99	0.86
SACS7	7.4	14.7	18.9	41.5	17.5	3.47	1.16	-0.59
SACS8	14.3	31.3	24.9	24.0	5.5	2.75	1.14	0.14
SACS9	8.3	16.6	21.7	35.9	17.5	3.38	1.19	-0.43
SACS10	34.6	31.3	24.0	7.8	2.3	2.12	1.05	0.65
SACS11	1.4	9.2	23.0	40.6	25.8	3.80	0.97	-0.56
SACS12	26.3	35.0	31.8	6.0	0.9	2.20	0.93	0.31
SACS13	14.7	33.6	37.3	11.5	2.8	2.54	0.97	0.24
SACS14	6.0	16.6	15.2	42.4	19.8	3.53	1.16	-0.60
SACS15	4.6	14.7	20.7	37.3	22.6	2.59	1.13	-0.53

*N* = 217; SACS, Staff Attitude to Coercion Scale.

### Exploratory factor analysis

**FIGURE 1:**
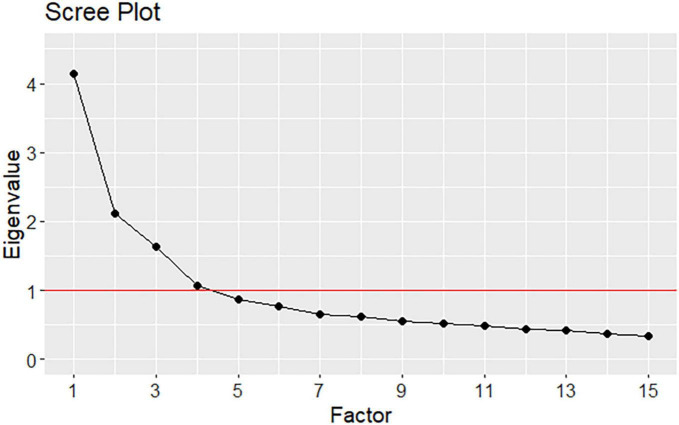
Scree plot of eigenvalues for the SACS.

The Bartlett sphericity score was statistically significant (*χ^2^* = 907.22, *df* = 105, *p* < 0.001), indicating that the correlation matrix significantly deviates from the identity matrix and, consequently, it is meaningful to conduct an EFA (52). The KMO-MSA value for the 15 items of the Italian version of the SACS was 0.80, meaning a meritorious sampling to perform EFA. Figure 1 displays Cattell’s scree plot of eigenvalues, which reveals four factors with *eigenvalues* higher than 1, the first *eigenvalue* being greater than four. Therefore, solutions including one, three and four factors were explored, as suggested by Cattell’s scree plot and by the literature, first, a single-factor solution of the SACS, second, the original structure consisting of three factors, and third, a four-factor solution. Inspection of the BIC values of the three estimated models (BIC_*one–factor*_ = −0.60, BIC_*three–factors*_ = −211.18, BIC_*four–factors*_ = −203.31) indicated that the one-factor solution was less acceptable than the three- and four-factor solutions. Moreover, the one-factor solution obtained an explained variance of 23% and factor loadings less than 0.40. Therefore, the one-factor solution was ruled-out. The three- and four-factor solutions resulted in an explained variance of 41% and 46%, respectively. Both the three- and four-factor solutions represented a good compromise between complexity and explained variance. However, the three-factor solution was slightly better than the four-factor structure, in that the former showed factor loadings higher than 0.40 compared to the latter. Of the total 41% explained variance of the three-factor model, the first, second, and third factors explained 19, 12, and 10% of the variance, respectively. Therefore, the three-factor structure was adopted due to its better overall performance and consistency with the theoretical framework of the original SACS. The three extracted factors were labeled following the terms of the original scale and according to their respective item content: *Factor 1* “Coercion as offending” (containing four items), *Factor 2* “Coercion as care and security” (seven items), and *Factor 3* “Coercion as treatment” (four items). More specifically, three items loaded on different factors with respect to the original scale, namely items 4 and 8 loaded on Factor 2 instead of Factor 1, and item 11 loaded on Factor 3 instead of Factor 2.

Table 2 shows the factor loadings of the three-factor model while Supplementary Table 2 displays the factor loadings of the discarded one- and four-factor solutions. Furthermore, the correlation matrix of the three factors is reported in Supplementary Table 3.

**TABLE 2:** Factor loadings for the three-factor model of the Italian version of the SACS.

	Three-factor model		
Items	Factor 1	Factor 2	Factor 3
SACS1	0.03	**0**.**69**	0.01
SACS2	0.19	**0**.**61**	0.10
SACS3	**0**.**46**	-0.34	0.10
SACS4	0.15	-**0**.60*	0.23
SACS5	-0.07	**0**.**55**	0.08
SACS6	-0.30	-0.05	**0**.**49**
SACS7	0.02	**0**.**58**	0.32
SACS8	0.28	-**0**.46*	0.20
SACS9	0.12	**0**.**42**	0.30
SACS10	-0.01	0.30	**0**.**42**
SACS11	-0.10	0.30	**0**.**42**
SACS12	-0.01	-0.08	**0**.**56**
SACS13	**0**.**47**	-0.40	0.03
SACS14	**0**.**68**	0.12	0.00
SACS15	**0**.**74**	0.02	-0.12

*N* = 217; *Items to be reversed; Factor 1 = Coercion as offending; Factor 2 = Coercion as care and security; Factor 3 = Coercion as treatment. Factors with significant loading (≥0.40) are reported in bold characters.

### Internal consistency of the three-factor model of the Italian version of the SACS

The internal consistency of the three-factor model of the Italian version of the SACS, assessed through Cronbach’s α, yielded acceptable indexes ranging from 0.64 to 0.77. More specifically, for Factor 1, “Coercion as offending,” α = 0.73 (95% CI = [0.66, 0.78]); for Factor 2, “Coercion as care and security,” α = 0.77 (95% CI = [0.70, 0.81]); last, for Factor 3, “Coercion as treatment,” α = 0.64 (95% CI = [0.54, 0.71]).

## Discussion and conclusion

The current study aimed to explore the factorial structure of the Italian SACS version among Italian staff members working in psychiatric wards. Our results confirmed the three-dimensional structure of the SACS, except for three items loading in different factors. More specifically, item 4, *“use of coercion is a declaration of failure on the part of the mental health services”* and item 8, *“coercion violates the patients’ integrity,”* originally part of factor 1, namely “Coercion as offending,” loaded on factor 2, namely “coercion as care and security”–notably, both reversed. These findings can suggest that in the professionals’ view that coercion measures are necessary for care and security reasons (pragmatic attitude, i.e., factor 2 “Coercion as care and security”) and they are not a violation of patient’s integrity neither as a failure of psychiatry services. In other words, it would seem that this pragmatic view justifies the use of coercion to such an extent that it overlooks both the possible harm to the patient and the possibility of favoring non-coercive measures whose non-application (or lack of success in terms of patient management) indicates a failure of the institutions of care.

Another difference regards item 11, *“use of coercion is necessary toward dangerous and aggressive patients,”* which loaded on factor 3, “Coercion as treatment,” rather than on factor 2 “Coercion as care and security.” This suggests that the use of coercive measures to manage dangerous or aggressive patients is viewed more from a therapeutic perspective (as a valid form of treatment), rather than a pragmatic one (as necessary for care and security reasons).

Notably, all the above-mentioned discussion are merely speculative and should be taken with caution, as they refer to the differences in the distribution of SACS item scores (factor structure) across different validation studies and could be due to the different nuances of items translation between languages. However, the use of coercion may be influenced by a “paternalistic culture” (i.e., a directive and authoritarian attitude based on the notions of benevolence and clinical expertise), as well as by individual beliefs (implicit and explicit) regarding social control, concepts of punishment, correction, and discipline (53–55). As these themes and concepts are also culturally determined, future cross-cultural studies are needed to clarify potential differences in staff attitudes toward coercion across different cultural contexts and geographical areas.

To our knowledge, the SACS has been also validated in Arabic (56), Polish (35), Japanese (36), German (24), and Indian languages (31). In this regard, the authors of the original SACS recently conducted a systematic review aimed at investigating the measurement properties of the SACS in different countries (30). Results showed that the scale has been successfully adapted to different cultural contexts, providing evidence for adequate structural validity and internal consistency across studies—other measurement properties (e.g., criterion validity, test-retest reliability) were poorly investigated and thus need to be better clarified. We can conclude that the Italian version of the SACS showed good internal consistency and structural validity, in line with previous validation studies; consequently, it proved to be a suitable tool to use in research and clinical settings.

Our study has several limitations. Indeed, we only recruited mental health professionals in one region in Central Italy, Lazio. The cultural and social heterogeneity of Italian regions could hinder the possibility to generalize the present results to the entire country. Generalizability was also limited by not including staff from other than GHPWs. However, staff form other wards are less involved in coercive measure providing, thus studying their attitudes would be beside the aims of the current study. Additionally, we could not test the construct (i.e., convergent, and divergent) validity of the SACS against a gold standard, due to the lack of other staff-attitudes-to-coercion tools (30). Last, we did not investigate the relationship between staff attitudes and the actual use of coercion, which, indeed, is an interesting future development of the present work. In this regard, semi-structured interviews would be carried out to assess and better frame the use of coercion by health care professionals.

Concluding, we assessed the factor structure of the Italian SACS version in a central Italian sample of healthcare professionals and found it to confirm the three-factor model of the original version as the best fit, with some few items migrating to a different factor. The characteristics of this self-rated instrument render it suitable for application in psychiatric ward contexts.

## Data availability statement

The raw data supporting the conclusions of this article will be made available by the authors, without undue reservation.

## Ethics statement

The study involved human participants who provided their written informed consent to participate in the study prior to participating in the survey.

## Author contributions

PV designed the study and conducted the data collection. PV and GK translated and adapted the questionnaire. GB conducted the statistical analyses. TB, PV, and GB drafted the manuscript. All authors reviewed, contributed, and approved the final version of the manuscript.
