# Supplementary material for: Psychometric properties of the Italian version of the staff attitude to coercion scale: an exploratory factor analysis
Source: Front Psychiatry. 2023 May 24;14:1172803. doi: 10.3389/fpsyt.2023.1172803 (PMC10244557; doi:10.3389/fpsyt.2023.1172803)
Supplement: Supplementary file 1 [file Data_Sheet_1.PDF]

Supplementary material to:

The Italian Version of the Staff Attitude to Coercion Scale: development and empirical validation

Paola **Venturini**[1], Giulia **Bassi**[2], Silvia **Salcuni**[2], Georgios **D**. **Kotzalidis**[3], Carla Ludovica **Telesforo**[1], Eleonora **Salustri**[1], Manuela **Trevisi**[1], Valentina **Roselli**[4], Lorenzo **Tarsitani**[4], Vittorio **Infante**[5], Cinzia **Niolu**[5], Gianmarco **Polselli**[1], Tommaso **Boldrini**[2]

[1] Department of Psychiatry, Rome ASL Roma 1, Rome, Italy

[2] Department of Developmental Psychology and Socialization, University of Padova, Padua, Italy

[3] NESMOS Department, Faculty of Medicine and Psychology, Sapienza University of Rome, Sant'Andrea University Hospital, Rome, Italy

[4] Department of Human Neurosciences, Faculty of Medicine and Dentistry, Sapienza University of Rome, Policlinico Umberto I, Rome, Italy

[5] Università di Roma Tor Vergata, U.O.C. Psichiatria e Psicologia Clinica, Policlinico Tor Vergata, Rome, Italy

paola.venturini@aslroma1.it

Index

Table S1. Items of the original English SACS and the adaptation of the Italian version of the SACS………..3

Table S2. Factor loadings of the one- and four-factor solutions of the Italian version of the SACS…………4

Table S3. Correlation matrix of the three factors of the Italian version of the SACS…………………………5

Table S1. Items of the original English SACS and the adaptation of the Italian version of the SACS.

Original scales	Items	English version	Italian translation
Coercion as offending (Factor 1)	3	Use of coercion can harm the therapeutic relationship.	L'uso della coercizione può danneggiare la relazione terapeutica.
	4	Use of coercion is a declaration of failure on the part of the mental health services.	L'uso della coercizione è una dichiarazione di fallimento da parte dei servizi di salute mentale.
	8	Coercion violates the patients' integrity.	La coercizione viola l'integrità dei pazienti.
	13	Too much coercion is used in treatment.	Viene utilizzata troppa coercizione nel trattamento.
	14	Scarce resources lead to more use of coercion.	Scarse risorse portano a maggior utilizzo di coercizione.
	15	Coercion could have been much reduced, giving more time and personal contact.	Si potrebbe ridurre molto la coercizione dedicando più tempo al paziente e al contatto personale.
Coercion as care and security (Factor 2)	1	Use of coercion is necessary as protection in dangerous situations.	L'uso della coercizione è necessario come protezione in situazioni pericolose.
	2	For security reasons, coercion must sometimes be used.	La coercizione va utilizzata qualche volta per motivi di sicurezza.
	5	Coercion may represent care and protection.	La coercizione può rappresentare cura e protezione.
	7	Coercion may prevent the development of a dangerous situation.	La coercizione può prevenire lo sviluppo di una situazione pericolosa.
	9	For severely ill patients, coercion may represent safety.	Per i pazienti gravemente ammalati, la coercizione può rappresentare sicurezza.
	11	Use of coercion is necessary toward dangerous and aggressive patients.	L'uso della coercizione è necessario verso pazienti pericolosi e aggressivi.
Coercion as treatment (Factor 3)	6	More coercion should be used in treatment.	Va utilizzata più coercizione nella terapia.
	10	Patients without insight require use of coercion.	I pazienti senza consapevolezza di malattia necessitano l'uso di coercizione.
	12	Regressive patients require use of coercion.	I pazienti con comportamenti regressivi necessitano dell'uso di coercizione.

Note. SACS = Staff Attitude to Coercion Scale.

Table S2. Factor loadings of the one- and four-factor solutions of the Italian version of the SACS.

	**One-factor model**	**Four-factor model**			
**Items**	**Factor 1**	**Factor 1**	**Factor 2**	**Factor 3**	**Factor 4**
SACS1	**0.61**	-0.03	**0.76**	-0.12	-0.08
SACS2	**0.47**	0.12	**0.66**	0.00	-0.01
SACS3	**-0.54**	0.32	-0.05	-0.01	**0.47**
SACS4	**-0.54**	0.01	-0.23	0.08	**0.56**
SACS5	**0.60**	0.00	0.31	0.15	***-0.34****
SACS6	***0.30****	-0.29	0.00	**0.45**	0.06
SACS7	**0.64**	0.04	**0.46**	0.32	-0.20
SACS8	**-0.50**	0.03	0.06	-0.03	**0.79**
SACS9	**0.42**	0.11	**0.36**	0.29	-0.09
SACS10	***0.31****	0.02	0.08	**0.69**	0.02
SACS11	**0.48**	-0.22	**0.52**	0.26	0.22
SACS12	***0.14****	0.06	-0.15	**0.66**	-0.04
SACS13	**-0.62**	**0.43**	-0.27	0.05	0.26
SACS14	***-0.26****	**0.71**	0.06	0.08	-0.02
SACS15	**-0.42**	**0.72**	0.02	-0.06	0.07

Note. N = 217; SACS = Staff Attitude to Coercion Scale; * = loadings that are below the cut-off value of 0.40.

Table S3. Correlation matrix of the three factors of the Italian version of the SACS.

	Factor 1	Factor 2	Factor 3
**Factor 1**	1.00	-0.29	0.01
**Factor 2**		1.00	0.20
**Factor 3**			1.00

Note. N = 217; Factor 1 = Coercion as offending; Factor 2 = Coercion as care and security; Factor 3 = Coercion as treatment.
